# A 8- year Bangladeshi girl with disseminated histoplasmosis, presented as chronic liver disease with portal hypertension: a rare case report

**DOI:** 10.1186/s12887-020-02189-4

**Published:** 2020-06-09

**Authors:** Luthfun Nahar, Md Benzamin, Naznin Sarkar, Urmi Roy, Kamrun Nahar, Md Rukunuzzaman, Khan Lamia Nahid, A. S. M. Bazlul Karim, Bishnu Pada Dey

**Affiliations:** Bangabandhu Sheikh Mujib Medical University, Dhaka, Bangladesh

**Keywords:** Child, Histoplasmosis, Portal hypertension

## Abstract

**Background:**

Histoplasmosis is a rare infectious condition with mainly pulmonary involvement. Disseminated histoplasmosis may occur in immunocompromised condition. It can present in different ways but jaundice and ascites is very uncommon.

**Case presentation:**

A 8- year old girl visited to department of pediatric gastroenterology & nutrition, Bangabandhu Sheikh Mujib Medical University, Dhaka, Bangladesh. Child presented with fever, jaundice and abdominal distension for 2 ½ months. There was no history of contact with tuberculosis patient and travelling to kala-azar, malaria endemic zone and no history of previous jaundice, blood or blood product transfusion, history of sib death, family history of jaundice or neuropsychiatric disorder, significant weight loss. On general examination she was fretful, febrile, moderately icteric, mildly pale, vitally stable, severely wasted and moderately stunted, skin survey revealed infected scabies, BCG vaccine mark was absent, generalized lymphadenopathy, hepato-splenomegaly and ascites present. After evaluating the physical findings, several investigations was done including lymphnode biopsy, then the case was finally diagnosed as Disseminated histoplasmosis with portal hypertension. Child was treated with injectable Deoxycholate Amphotericin B for 28 days and improved on follow up.

**Conclusion:**

We suggest that children presenting with fever, jaundice, lymphadenopathy and hepatosplenomegaly and portal hypertension, disseminated histoplasmosis can be one differential.

## Background

Histoplasmosis was first described over a century ago by American physician Samuel Darling. It is primarily a pulmonary disease and soil is the environmental reservoir. It exist as mold in the environment. Two types of conidia as macroconidia (8–15 μm) and microconidia (2–4 μm) are formed. It occurs commonly in North and Central America but may presents in other parts around the world. Exposure to histoplasma is common in the population of endemicity, but symptomatic infection is uncommon [1]. Pulmonary infection occurs by inhalation of microconidia, it may present as acute or chronic pulmonary histoplasmosis. Patient may develope cough, sputum production, hemoptysis, dyspnea. Disseminated histoplasmosis may presents in various form, so difficult to diagnose. It experience asymptomatic hematogenous dissemination through the reticuloendothelial system via parasitized macrophages. The symptoms include fever, malaise, anorexia, weight loss. On physical findings patient may have anemia, lymphadenopathy, petechiae, mucous membrane ulceration, hepatosplenomegaly etc.

Disseminated histoplasmosis can present in different ways but jaundice and ascites is very uncommon. Disseminated form is common in immune compromised condition as infant, older age, HIV infection, transplant recipient, hematological malignancy or those on corticosteroids or TNF-α inhibitors therapy which are associated with increased risk of opportunistic infections. Disseminated form is the commonest presentation in infant and toddlers. Among this age group 60–80% patients has acute disseminated progressive disease [2].

We are reporting this case because our patient had strongly features of chronic liver disease with portal hypertension and another differential was disseminated tuberculosis. But finally she diagnosed as a case of disseminated histoplasmosis. This case emphasizes the need to keep disseminated histoplasmosis in differential diagnosis of children presenting with fever, jaundice, lymphadenopathy and hepatosplenomegaly.

## Case presentation

**Fig. 1:**
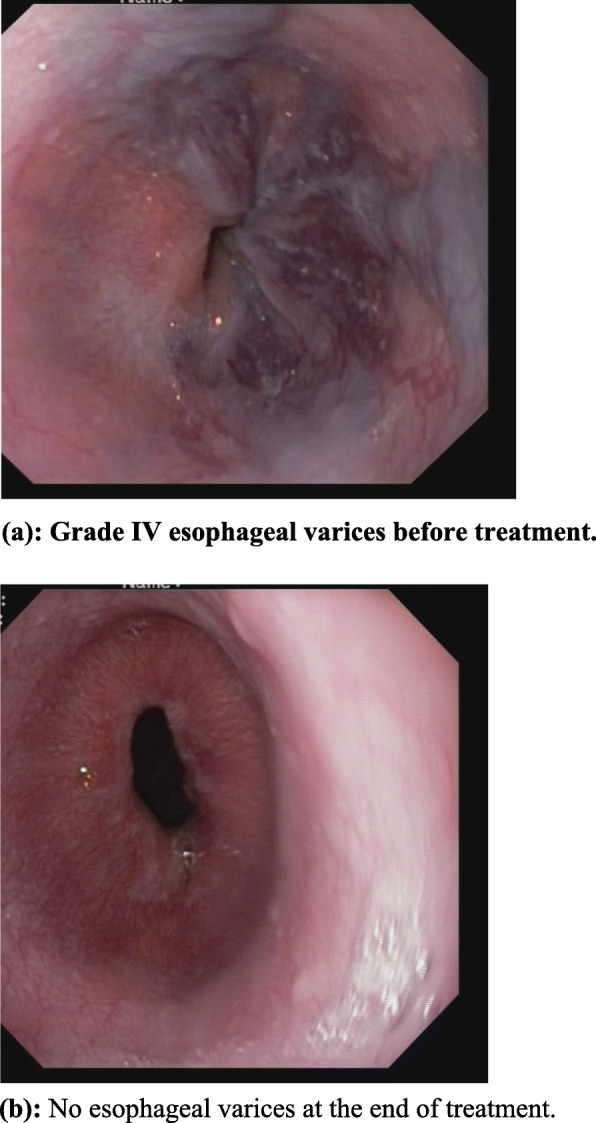
(**a**) Grade IV esophageal varices before treatment. (**b**) No esophageal varices at the end of treatment

**Fig. 2:**
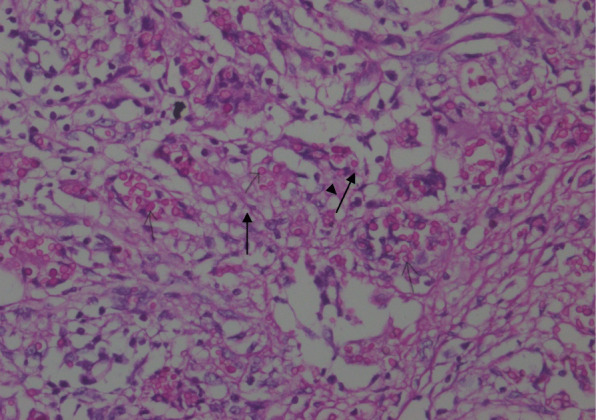
The arrow mark is showing *Histoplasma capsulatum*

A 8 year old girl of non consanguineous parents from low socioeconomic condition, immunized as per EPI schedule got admitted with fever, jaundice and abdominal distension. Fever was for 2 & ½ months, high grade, intermittent, usually developed at evening and night, highest recorded temperature was 104 °F, subsided after taking antipyretic and not associated with chills and rigor. She had also jaundice for 2 months followed by abdominal distension. There was no history of contact with tuberculosis patient and travelling to kala-azar, malaria endemic zone and no history of previous jaundice, blood or blood product transfusion, history of sib death, family history of jaundice or neuropsychiatric disorder, significant weight loss. For these she was treated initially by different injectable antibiotics in medical college hospital but didn’t improve. On general examination she was fretful, febrile, moderately icteric; mildly pale, vitally stable, severely wasted and moderately stunted and developmentally age appropriate, skin survey revealed infected scabies, BCG vaccine mark was absent, generalized lymphadenopathy present, maximum 1.5 × 1.5 cm at right anterior cervival group, non tender, discrete, movable, not fixed with underlying structure and overlying skin, bony tenderness absent and there was no stigmata of chronic liver disease. Abdomen was distended, hepatomegaly about 7 cm, spleen enlarged about 6 cm, ascites present. Other system examination revealed normal findings. Slit lamp examination of eye showed no Kayser Fleisher ring. Complete blood count revealed mild anemia with neutrophilic leukocysis and thrombocytosis, ESR was very high (130 mm), Mantoux test was negative, ICT for kala-azar and malaria was negative, liver function showed direct hyperbilirubinemia, slightly low albumin, normal serum alanine aminotransferase and normal prothrombin time, ascitic fluid study had high lymphocyte count, atypicallymphocytes with high SAAG, HBsAg, antibody for Hepatitis C virus, antibody to smooth muscle antibody, antibody to liver kidney microsomal antibody, anti neuclear antibody were negative. Total Immunoglobulin G, chest X-ray and bone marrow study was normal. Lactate dehydrogenase was high. Ultrasonography of whole abdomen showed hepatomegaly with coarse hepatic parenchymal echotexture, splenomegaly with enlarged hilar vessels, enlarged lymph nodesat porta hepatis and around the celiac plexus, mildly dialated intrahepatic biliary tree and common bile duct is mildly dialated. Endoscopy revealed grade IV esophageal varices; Fig. 1a. Finally lymph node biopsy was done and histopathology report showed large number of foreign body giant cells and small ill defined granuloma. Numerous histoplasma capsulati spores within giant cells, histocytes and extracellular space are seen. Histoplasma capsulati spores are PAS stain positive (Fig. 2).

After evaluating the clinical data, physical findings and investigation results, the case was finally diagnosed as Disseminated histoplasmosis with portal hypertension.

We started treatment after counseling the parents with injectable Deoxycholate Amphotericin B(1 mg/kg/day) for 28 days, multivitamin, 5% Permethrin cream for scabies, endoscopic variceal ligation for portal hypertension. There was no drug related or infusion related complication developed. During treatment period her clinical condition was dramatically improved. She became afebrile, regaining of apettite, jaundice was declining, organomegaly reduced. We discharged her with oral Itraconazole (5–10 mg/kg/day), Tab. Propranolol and multivitamin. Itraconazole was given for 5 months and propranolol for 1 month. After 1 month of treatment follow up endoscopy showed no esophageal varices. During treatment we follow up the patient regularly based on clinical and biochemical parameters. At the end of treatment her weight increased, jaundice disappeared; lymphadenopathy present but not significant, hepatomegaly was 3 cm and there was no splenomegaly and no ascites. There was no esophageal varices on follow up endoscopy; Fig. 1b.

Follow up ultrasonography was done after 1 month at the end of treatment with Amphotericin B. Report showed hepatomegaly with coarse echopattern, normal biliary tree. Splenomegaly with enlarged hilar vessels. No ascites.

## Discussion and conclusion

Histoplasmosis is a systemic fungal disease caused by soil based dimorphic fungus *Histoplasma capsulatum*. Most of this infections are asymptomatic or self limiting. On the basis of clinical features there are four types of histoplasmosis as asymptomatic, acute pulmonary, chronic pulmonary and disseminated. A very few patients result in severe and progressive dissemination and it is the rarest form. The most common symptoms of the disseminated forms are fever (89.1%), respiratory symptoms (38.1%), and weight loss (37.4%) and common sign includes splenomegaly (72%), hepatomegaly (68.1%) and lymphadenopathy (41.2%) [3]. The presenting case also had the similar manifestations as fever, weight loss, lymphadenopathy, splenomegaly and hepatomegaly. About 10% of patients present as disseminated histoplasmosis. Initial infection is in lung alveoli, then it is transformed by reticuloendothelial system and spread to various organ which results progressive disseminated histoplasmosis. The clinical feature depends on size of inoculum, underlying health and immune status. In immunecompetent host it is usually asymptomatic and self limiting. In immunecompromissed condition, all organ and tissue may be involved and in HIV infection, it is usually disseminated form and typically seen when CD4+ cell count is less than 200/cmm [4]. In our patient disseminated histoplasmosis involved the lymph node, liver, spleen. Obstruction by periportal lymph nodes results obstructive jaundice. The high SAAG ascites may be due to chronic parenchymal liver disease resulting from histoplasma induced liver disease.

In endemic areas, histoplasmosis can be mistaken for tuberculosis during diagnosis, since the clinical manifestations are very similar, and it is not easy to differentiate between the two diseases, especially in patients with AIDS. Sometimes in these endemic areas, histoplasmosis is a primary consideration in HIV-infected patients with suspected tuberculosis [5].

Here is a case report of disseminated histoplasmosis, a 3 ½ years old Bangladeshi boy, presented with low grade fever, recurrent multiple abscess with generalized lymphadenopathy, anaemia, hepatomegaly, osteolytic lesion in bone and initially diagnosis was Tuberculosis. Our case also present with fever, generalized lymphadenopathy, anaemia and hepatomegaly [6].

The infection may also involve adrenal glands, gastrointestinal tract which results ulceration, bone marrow that leads to pancytopenia, the central nervous system etc. On rare occasion it may associated with hypercalcemia and this is due to increased 1, 25 dihydroxyvitamin D production from the fungal granulomas [5]. Diagnosis of disseminated histoplasmosis is difficult because of various form of manifestation. The clinical features are very similar to tuberculosis and very difficult to differentiate. Histoplasma detection can be done by growth in culture, fungal stains (Bone marrow aspirate, peripheral blood smear, lymph node biopsy, bronchoalveolar lavage fluid, transbrochial biopsy specimen and biopsy from cutaneous lesions), serologic tests involving detection of antigen and antibodies, PCR and DNA assay for *Histoplasma capsulatum* [7]. Use of Histoplasma antigen in urine for rapid diagnosis of suspected disseminated histoplasmosis can be done [8].

In our patient we confirmed diagnosis by histopathological detection of *Histoplasma capsulatum* in lymph node tissue.

Mildly symptomatic patient may be treated by oral Itraconazole or Fluconazole, but Itraconazole is superior. Severe form of disease should be treated with injectable Amphotericin B 1 mg/kg/day for 4–6 weeks followed by oral Itraconazole 5–10 mg/kg/day in two devided dose for 6–12 months [9]. The mortality rate is high inspite of antifungal treatment [10]. In this case we have treated with injectable Deoxycholate Amphotericin B for 28 days followed by oral Itraconazole for 5 months and the patient improved at the end of treatment.

Though disseminated histoplasmosis is not so common in Bangladesh. The presentation are variable and diagnosis is very important for specific treatment. So, it should be kept in differential diagnosis of children presenting with fever, jaundice, lymphadenopathy and hepatosplenomegaly.

## Patient perspective

Parents said that “we are happy as our baby is improved and thanks to all doctors.”

## Data Availability

The datasets used and/or analysed during the current study available from the corresponding author on reasonable request.

## Abbreviations

BCG: Bacillus Calmette–Guérin
HIV: Human immunodeficiency viruses
EPI: Expanded Program on Immunization
SAAG: Serum Ascites Albumin Gradient
PAS: Periodic acid–Schiff
ESR: Erythrocyte Sedimentation Rate
TNF: Tumor necrosis factor
PCR: Polymerase chain reaction
